# MIRPIPE: quantification of microRNAs in niche model organisms

**DOI:** 10.1093/bioinformatics/btu573

**Published:** 2014-08-26

**Authors:** Carsten Kuenne, Jens Preussner, Mario Herzog, Thomas Braun, Mario Looso

**Affiliations:** ^1^Group of Bioinformatics and ^2^Cardiac Development and Remodelling, Max Planck Institute for Heart and Lung Research, Ludwigstrasse 43, D-61231 Bad Nauheim, Germany

## Abstract

**Summary:** MicroRNAs (miRNAs) represent an important class of small non-coding RNAs regulating gene expression in eukaryotes. Present algorithms typically rely on genomic data to identify miRNAs and require extensive installation procedures. Niche model organisms lacking genomic sequences cannot be analyzed by such tools. Here we introduce the MIRPIPE application enabling rapid and simple browser-based miRNA homology detection and quantification. MIRPIPE features automatic trimming of raw RNA-Seq reads originating from various sequencing instruments, processing of isomiRs and quantification of detected miRNAs versus public- or user-uploaded reference databases.

**Availability and implementation:** The Web service is freely available at http://bioinformatics.mpi-bn.mpg.de. MIRPIPE was implemented in Perl and integrated into Galaxy. An offline version for local execution is also available from our Web site.

**Contact:** Mario.Looso@mpi-bn.mpg.de

**Supplementary information:** Supplementary data are available at *Bioinformatics* online.

## 1 INTRODUCTION

MicroRNAs (miRNAs) are ∼22 nucleotides long and belong to the class of snRNAs. miRNAs serve numerous roles in downregulation (transcript degradation and sequestering, translational suppression) of gene expression. In general, miRNAs are assumed to regulate multiple targets although effects on most targets are relatively mild (Ameres and Zamore, 2013). Isoforms of miRNAs resulting from imperfect digestion by Drosha and Dicer or RNA editing by specialized enzymes represent a challenge during the determination of correct read counts following RNASeq. miRNA variants might be ‘silent’ (3′ modification = isomiR) or target different mRNAs when changes occur in the 5′ regions responsible for complementary binding. Sequence differences between taxa hamper quantification, especially if no genomic or miRNA data for the studied organism are available as in the case of niche model organisms. Sequencing errors can further complicate the identification of miRNAs. These effects should ideally be addressed on multiple levels, including (i) isomiR handling, (ii) enforcement of a minimum read copy number, (iii) clustering of similar miRNAs, (iv) removal of relatively low abundance reads and (v) optional fallback to the miRNA family level. A set of applications in the field attempts to cover these features, but a Web-based tool able to unify all functionalities that can be applied to any organism is critically missing (An *et al.*, 2013; Giurato *et al.*, 2013; Wen *et al.*, 2012).

## 2 WORKFLOW AND FEATURES

MIRPIPE uses open-source binary tools including the FASTX-Toolkit (Pearson *et al.*, 1997), Cutadapt (Martin, 2011) and BLASTN (Boratyn *et al.*, 2013) for data processing. The pipeline was integrated into a Galaxy-based Web platform (Goecks *et al.*, 2010) but is also available for download and local execution. A detailed explanation of the algorithm can be found in Supplementary File S1.

The workflow starts with the upload of a compressed FASTQ/FASTA read file using the Web interface or the MIRPIPE FTP server. MIRPIPE can fully process raw reads originating from Illumina, 454, IonTorrent or Sanger sequencing instruments including adapter trimming. A reference FASTA database bearing mature target miRNAs can either be selected from current miRBase release (Griffiths-Jones *et al.*, 2006) or can be uploaded by the user.

The raw reads are processed to optionally remove an adapter sequence and trim for a minimum quality (default Q20). Only reads of the desired size range are selected to limit the pool to mature miRNAs. Duplicate reads are collapsed to decrease the number of necessary homology searches, and only those sequences represented by a minimum count are kept for further analyses. This measure is intended to remove unique reads, which frequently denote sequencing errors or miRNA variations that are expressed near to the detection limit, preventing reliable quantification.

Read counts from isomiRs of the same miRNA are combined. These isomiR read sequences may only differ by the 3′ end and are thus putatively encoded by the same gene. Only one nucleotide may differ between two sequences to be counted as isoforms of the same miRNA, and only the longest sequence is used in the next step to further reduce the amount of homology searches.

The remaining read sequences are used for a sequence similarity search versus the chosen reference database of miRNAs.

Mature reference miRNAs and their precursors are optionally collated by name on the family level to remove redundancy introduced by organism prefixes and precursor suffixes (e.g. bta-miR-200a, oan-miR-200a-3p > miR-200a).

For each read, the detected reference miRNA families are scored based on the minimum number of mismatches. If a read matched equally well versus multiple miRNA families, the respective families are joined by single linkage clustering. This permits the inclusion of reads that cannot be matched uniquely, as well as the exact measurement of the fraction of ambiguously matching reads and thereby the reliability of the match. By default, only those read sequences that are at least 5% as abundant as the most abundant sequence per miRNA family cluster are denoted to reduce the impact of sequencing errors and increase robustness.

Counts per miRNA family and cluster are presented for download. Currently, MIRPIPE can complete a job within 0.5–2 h, depending on the file size and the selected reference database. MIRPIPE quantification results can be directly used for differential expression analysis using other tools on our Web site (Supplementary File S1).

## 3 BENCHMARK

To demonstrate congruent results for MIRPIPE, we compared the results with an miRNA analysis based on a genomic mapping of Illumina HiSeq reads (Lawless *et al.*, 2013). We identified 96% of the published miRNAs (Supplementary File S2). Furthermore, we compared our tool with a similar approach without the need for a genome sequence by analyzing a public dataset (Zhang *et al.*, 2013) with the CLC Genomics Workbench. In this case, 84% of the miRNAs were identical (Supplementary File S2).

**Fig. 1.**
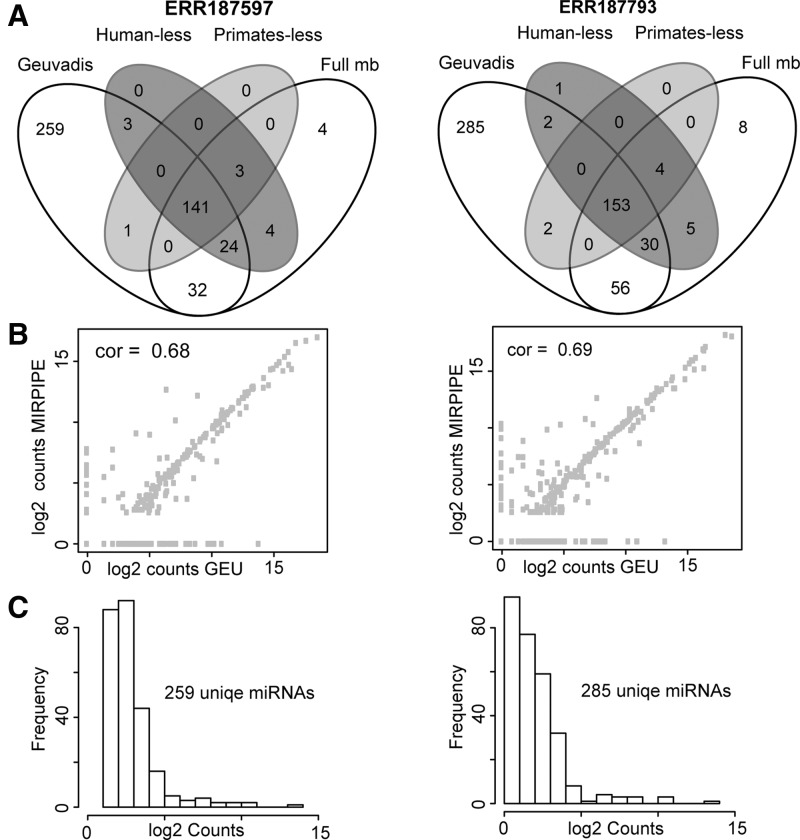
**A**) Comparison of MIRPIPE prediction on two gold standard (GS) datasets using full miRBase and reduced miRBase as reference set. (**B**) Spearman correlation of absolute counts of GS and MIRPIPE. (**C**) The large number of GS-specific miRNA identifications is caused by low counts, filtered out by MIRPIPE default parameters

Finally, we checked the predictive efficiency of our tool for niche models based on a human RNA-Seq dataset (Lappalainen *et al.*, 2013). Here, we performed MIRPIPE versus a reference database bearing (i) the complete miRBase, (ii) miRBase excluding human miRNAs and (iii) miRBase excluding miRNAs of all primates. The absence of closely related reference sequences resulted in only a marginal loss of sensitivity for MIRPIPE, indicating its aptitude for the analysis of niche model organisms (Fig. 1, Supplementary File S2).

*Funding*: Excellence Cluster Cardio-Pulmonary System (ECCPS); MPI for Heart and Lung Research.

*Conflict of interest*: none declared.

## Supplementary Material

Supplementary Data
