# Supplementary material for: MIRPIPE: quantification of microRNAs in niche model organisms
Source: Bioinformatics. 2014 Aug 26;30(23):3412–3. doi: 10.1093/bioinformatics/btu573 (PMC4816158; doi:10.1093/bioinformatics/btu573)
Supplement: Supplementary Data [file supp_30_23_3412__index.html]

MIRPIPE – quantification of microRNAs in niche model organisms — MIRPIPE: quantification of microRNAs in niche model organisms — MIRPIPE: quantification of microRNAs in niche model organisms — Supplementary Data 

# MIRPIPE: quantification of microRNAs in niche model organisms

## Supplementary Data

files

**Files in this Data Supplement:**

- Supplementary Data - pdf file
- Supplementary Data - pdf file
