# Supplementary material for: MIRPIPE: quantification of microRNAs in niche model organisms
Source: Bioinformatics. 2014 Aug 26;30(23):3412–3. doi: 10.1093/bioinformatics/btu573 (PMC4816158; doi:10.1093/bioinformatics/btu573)
Supplement: Supplementary Data [file supp_btu573_mirpipe_supplementary_1.pdf]

CONTENTS

1. Overview and Objective........1

2. Workflow........2

2.1 Upload sequence reads........2

2.2 Select / Upload reference database of miRNAs........2

2.3 Optionally trim reads for adapter sequence and quality, filter for length........3

2.4 Collapse duplicate reads and optionally remove low abundance reads........3

2.5 Cluster isomiRs........4

2.6 Homology search versus reference DB........4

2.7 Collate miRNA on family level........4

2.8 Cluster miRNAs by ambiguously matching reads and optionally remove comparatively low abundance reads........4

2.9 Assemble quantification data........5

3. Output Files........5

4. Example 1: Output interpretation........6

5. Example 2: From raw data to differential expression (DESeq2)........7

6. Contact........8

1. OVERVIEW AND OBJECTIVE

A host of applications exist in the field of RNA-Seq miRNA analyses, which attempt to quantify transcripts based on reference genomes or reference miRNAs, using different degrees of granularity including sub-sequences and precursors of miRNAs, deviating handling of technical and biological variation, offering a range of interfaces to the user (web, command line, graphical user interfaces), and bearing variable demands considering user expertise in informatics and biological interpretation.

MIRPIPE offers the following unique combination of features:

- Robust identification of miRNAs able to deal with comparisons spanning multiple species necessary for cases without a reference genome or previously identified miRNAs in the examined organism
- The option to include a user-supplied reference database of miRNAs
- Proper handling of biological variation (isomiRs, cluster of related miRNAs)
- Proper handling of technical variation (i.e. sequencing errors) introduced by 454 or IonTorrent sequencers

- Output of one count value per identified miRNA or miRNA family able to directly serve as input for differential expression analyses
- Interfaces for both use cases: quick and simple online analyses as well as inclusion in Linux workflow scripts
- Freeware and Open Source

MIRPIPE is specifically tuned to be robust versus technical variation (i.e. errors) introduced by sequencing technologies like 454 or IonTorrent, as well as biological variation due to sequence differences originating from the evolutionary distance between query and reference species. These effects are addressed on multiple levels (isomiR handling, minimum read copy number, clustering which removes comparatively low abundance reads, centring on the miRNA family).

2. WORKFLOW

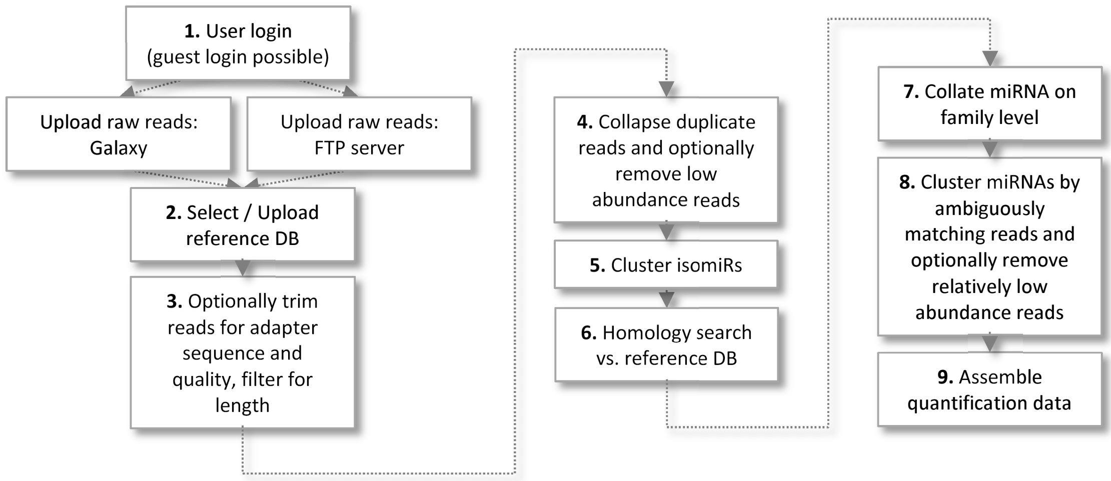

2.1 Upload sequence reads

The user can upload either FASTQ or FASTA files bearing reads using our MIRPIPE server web interface directly (Get Data / Upload File) or the MIRPIPE FTP server (Get data/ Upload File). These should ideally be compressed (.zip, .gz) to reduce upload time. The pipeline can fully process raw reads originating from Illumina, 454, IonTorrent or Sanger sequencing instruments including adapter trimming.

Parameters

Reads
File bearing reads in FASTQ or FASTA format (ideally zip compressed). This file can either be uploaded using the Galaxy Upload Tool (Helpful Tools / Get Data / Upload Files) or using an account on our FTP server. The latter is only possible after user registration, which automatically creates an account with the same username (=email) and password on the FTP server (ftp://bioinformatics.mpi-bn.mpg.de/). After uploading to the FTP the files have to be uploaded to the MIRPIPE server (Helpful Tools / Get Data / Upload Files). The data will be deleted from the server after two weeks.

2.2 Select / Upload reference database of miRNAs

A reference FASTA database bearing mature target miRNAs can either be selected from the pre-processed current miRBase release 20 data harbouring 30424 entries of 206 species or can be uploaded by the user in FASTA format. The user can optionally choose a subset of the miRBase reference miRNAs bearing only miRNAs of the desired organism to limit the comparison to the e.g. closest relative. If the chosen reference FASTA file does not obey to the naming convention of miRBase (<species>-miR-<#>-<suffix>), the "family name clustering" parameter should be turned off.

Parameters

Reference database
Preprocessed DBs (full miRBase or miRNAs of only one species) or any user uploaded FASTA file bearing mature miRNAs. The correct miRBase file can be downloaded for offline usage: ftp://mirbase.org/pub/mirbase/CURRENT/mature.fa.gz.

2.3 Optionally trim reads for adapter sequence and quality, filter for length

The raw read data is then processed to optionally remove an adapter sequence and trim according to a quality threshold (default Q>=20). Only reads of the desired size range are selected to limit the pool to likely mature miRNAs (default: 18-28 nt).

Parameters

Adapter sequence
Nucleotide sequence of the adapter to be removed from the 3' end using Cutadapt. By default the larger of the following values is used as the maximum mismatch number: 1, 10% of the adapter length. These values can be changed inside the mirpipe.pl script.

Minimum read length: 18
Minimum length of a read after trimming to be considered in the analysis.

Minimum base quality: 20
Minimum PHRED quality for FASTQ data. Nucleotides with lower quality will be trimmed. This parameter is not used if FASTA formatted read data is supplied.

Maximum read length: 28
Maximum length of a read to be considered in analysis.

2.4 Collapse duplicate reads and optionally remove low abundance reads

Duplicate reads are collapsed to decrease the number of necessary homology searches (the number of duplicates per read is noted). Only those sequences present a minimum number of times (default = 5) are kept for further analyses. This measure is intended to remove unique reads which frequently denote sequencing errors or lowly expressed miRNAs that can't be reliably quantified. Setting this parameter to "1" will increase sensitivity.

Parameters

Minimum read copy number: 5
A read sequence must be present at least this number of times to be included.

2.5 Cluster isomiRs

Read counts from isomiRs of the same miRNA are combined. These isomiR read sequences may only differ by the 3' end and are thus putatively encoded by the same gene and bear the same target specificity. This function allows the summary of putatively functionally equivalent isomiRs resulting from imperfect digestion by the RNases Drosha and Dicer or RNA-Editing by specialized enzymes resulting in 3' modification. Only the final 3' nucleotide may differ between two sequences to be counted as isoforms of the same miRNA and only the longest isoform sequence is used in the next step to reduce the amount of homology searches per miRNA.

2.6 Homology search versus reference DB

The resulting read sequences are compared versus the chosen reference database of miRNAs. Sensitivity and specificity of this BLASTN homology search can be controlled using various parameters inside the mirpipe.pl script. Parameters are optimized for small query sequences (-num_alignments 15 -word_size 7 -evalue 10 -dust no -strand plus). The resulting hits are filtered to exclude those with too many mismatches ((read length - alignment length) + mismatches + gaps = final mismatches).

Parameters

Maximum mismatches: 4
Maximum number of mismatches allowed between reference miRNA and read sequence ((read length - alignment length) + mismatches + gaps = final mismatches). This parameter controls the size of the miRNA clusters: more mismatches allowed = larger clusters.

2.7 Collate miRNA on family level

Mature miRNAs and their precursors are optionally collated by name on the family level to remove redundancy (ex. bta-miR-200a,oan-miR-200a-3p,tgu-miR-200a-3p -> miR-200a). Otherwise the complete miRNA names given in the reference database are carried over resulting in more detailed but also more redundant output. Turning off the family name clustering can be advisable in case the reference database of miRNA sequences does not obey to the naming convention of miRBase (<species>-miR-<#>-<suffix>).

Parameters

Family name clustering: Yes
Collapse the names of all variants of a miRNA to the miRNA family (ex. bta-miR-200a,oan-miR-200a-3p,tgu-miR-200a-3p -> miR-200a).

2.8 Cluster miRNAs by ambiguously matching reads and optionally remove comparatively low abundance reads

Detected reference miRNA families per read are scored based on the minimum number of mismatches. If a read matched equally well versus multiple miRNA families, the respective families are joined by single linkage clustering. By default only those read sequences that are at least 5% as abundant as the most abundant sequence per miRNA family cluster are denoted (ex. most abundant sequence = 100 reads, cut-off = 5 reads). This is intended to further suppress reads resulting from sequencing errors or biological miRNA variations that are expressed close to the detection limit.

Parameters

Minimum cluster abundance: 5%
Remove read sequences from a cluster that are less than x% as abundant as the most abundant sequence. This is intended to suppress reads resulting from sequencing errors or biological miRNA variations that are expressed near the detection limit. This parameter controls the size of the miRNA clusters: lower minimum cluster abundance = larger clusters.

2.9 Assemble quantification data

In order to obtain one count value per miRNA, miRNA family clusters are finally split to separate each element. If a read matched multiple miRNAs equally well, they are counted fully for all of the respective miRNAs. This can lead to a situation where the summarized read counts of all miRNAs can be higher than the amount of reads totally matching. Therefore each miRNA is associated with an ambiguity value, denoting the share of reads that could not be placed clearly (e.g. 11/89 reads ambiguous = 0.12). If this value is high, the respective miRNA count may be misleading. Finally, the most abundant sequence matching a miRNA is given (primary sequence) as well as the number of reads matching it.

3. OUTPUT FILES

mirpipe_cluster.tsv: MIRPIPE miRNA clusters = output of one read sequence per line

This file is centred on the different read sequences found per miRNA cluster that result from biological and technical variation. Only those read sequences that are >=5% as abundant as the most abundant sequence per cluster are denoted by default. If a read matched equally well versus multiple miRNAs, the respective miRNAs or miRNA clusters are joined by single linkage clustering.

```
Columns:
Cluster          Cluster number
Sequence         Read sequence
Count            Summarized read count for all duplicates of this read
miRNA            Name of miRNA or miRNA families

Example (sorted for cluster number, count):
Cluster  Sequence                  Count    miRNA
90       CAGTACTGTGATAACTGAAGAA    33       miR-101a
90       CTACTGTGATAACTGACT        17       miR-101c,miR-101a
```

mirpipe_cluster.fasta: MIRPIPE cluster sequences

All sequences reported in the MIRPIPE miRNA cluster file in FASTA format.

```
>miR-101a count=33
CAGTACTGTGATAACTGAAGAA
>miR-101a,miR-101c count=17
GTACTGTGATAACTGACT
```

mirpipe_mirna.tsv: MIRPIPE miRNAs = output of one miRNA per line

This file includes one count value per miRNA and can directly serve as input for subsequent differential expression analyses. It is based on clusters of highly similar miRNAs, where a clear assignment of reads is not always possible, since the same read can match equally well to multiple reference miRNAs. Only those miRNA sequences are reported that are >5% as abundant as the most abundant sequence in its cluster.

```
Columns:
miRNA                     Name of miRNA or miRNA family
Count                     Summarized read count including isomiRs, biological/technical sequence variations
Ambiguity                 Ratio of reads that mapped equally well to other miRNAs inside the cluster
Cluster                   miRNA family cluster number
Primary sequence          Most abundant sequence for this miRNA inside the cluster
Primary sequence count    Count of the most abundant sequence for this miRNA inside the cluster
Cluster members           A comma-separated list of all members of the miRNA family cluster

Example (sorted for cluster number, expression):
miRNA     Expression  Ambiguity  Cluster  Primary Sequence      PS Reads  Cluster members
miR-101a         143       0.12       90  CAGTACTGTGATAACTGAAGAA      33  miR-101a,miR-101c
miR-101c          17          1       90  GTACTGTGATAACTGACT          17  miR-101a,miR-101c
```

4. EXAMPLE 1: OUTPUT INTERPRETATION

The following example shows a MIRPIPE result using default parameters. Two miRNAs (miR-2478,miR-3968) were joined into a miRNA cluster based on BLASTN results.

```
mirpipe_cluster.tsv:
Cluster  Sequence                  Count    miRNA
192      ATCCCACTTCTGACACCA        69       miR-2478
192      ATCCCACTCTCAACACCA        11       miR-3968
192      ATCCCACTCCTGACACCA        11       miR-2478,miR-3968
192      ATCCCATTCTTGACACCA        9        miR-2478
192      TCGAATCCCACTCCTGACACCA    6        miR-3968
192      AATCCCACTCTCAACACCA       5        miR-3968
192      TCAAATCCCACTCTCAACACCA    5        miR-3968

mirpipe_cluster.fasta:
>miR-2478 count=69
ATCCCACTTCTGACACCA
>miR-3968 count=5
AATCCCACTCTCAACACCA
>miR-3968 count=11
ATCCCACTCTCAACACCA
>miR-2478,miR-3968 count=11
ATCCCACTCCTGACACCA
>miR-2478 count=9
ATCCCATTCTTGACACCA
>miR-3968 count=5
TCAAATCCCACTCTCAACACCA
>miR-3968 count=6
TCGAATCCCACTCCTGACACCA

mirpipe_mirna.tsv:
miRNA    Count    Ambiguity        Cluster  Primary Sequence  Primary Sequence Count    Cluster members
miR-2478 89       0.12             192      ATCCCACTTCTGACACCA        69               miR-2478,miR-3968
miR-3968 38       0.29             192      ATCCCACTCTCAACACCA        11               miR-2478,miR-3968
```

The mirpipe_cluster.tsv file depicts the best BLASTN hit per read sequence based on the least number of mismatches. Sequences are sorted for expression from top to bottom with the least expressed sequence still at least 5% as abundant as the most expressed sequence (69 <> 5). The two miRNAs were joined to a cluster because one of the read sequences showed a BLASTN hit which fit equally well to both reference sequences (192 ATCCCACTCCTGACACCA 11 miR-2478,miR-3968). If another query had found that e.g. miR-2478 and miR-1000 had resulted in equally similar homologies, the two clusters would have been joined to miR-2478,miR-3968,miR-1000.

The mirpipe_cluster.fasta file shows all read sequences found in mirpipe_cluster.tsv converted to FASTA format.

The mirpipe_mirna.tsv file attempts to include one count value per miRNA in order to simplify later quantification. The count values for each sequence detected per miRNA are summarized (e.g.: miR-2478 = 69 + 11 + 9 = 89, miR-3968 = 11 + 11 + 6 + 5 + 5 = 38). Since some of the reads matched two different miRNAs equally well (miR-2478,miR-3968 = 11), these reads are counted fully for both miRNAs. This leads to a situation where the summarized read counts of all miRNAs can be higher than the amount of reads totally matching. Each miRNA is associated with an ambiguity value, denoting the share of reads that could not be placed clearly (e.g. miR-2478: 11/89 ambiguous = 0.12). If this value is high, the respective miRNA count may be misleading. Finally, the most abundant sequence matching a miRNA is given (primary sequence) as well as the number of reads matching it.

5. EXAMPLE 2: FROM RAW DATA TO DIFFERENTIAL EXPRESSION (DESEQ2)

To run differential expression analysis on samples processed by MIRPIPE, we will use basic tools already included in The Galaxy based MIRPIPE Server to build up a count table, which serves as a good starting point for further analyses.

1. Upload all samples and possible replicates to the MIRPIPE server and process them individually with the MIRPIPE tool.

2. Use the Column Join tool to join MIRPIPE results into one matrix file per desired sample pair to be compared (tab-delimited: miRNA count1 count2 …). To do so, use a MIRPIPE_mirna.tsv file as the first file for the join and include both columns (c1,c2). To fill missing values in other datasets, set *Fill empty columns* to Yes and *Fill Columns By* to a single fill value (since it's count data, we recommend taking zero (0), see Figure 1). Use all other data sets as additional inputs.

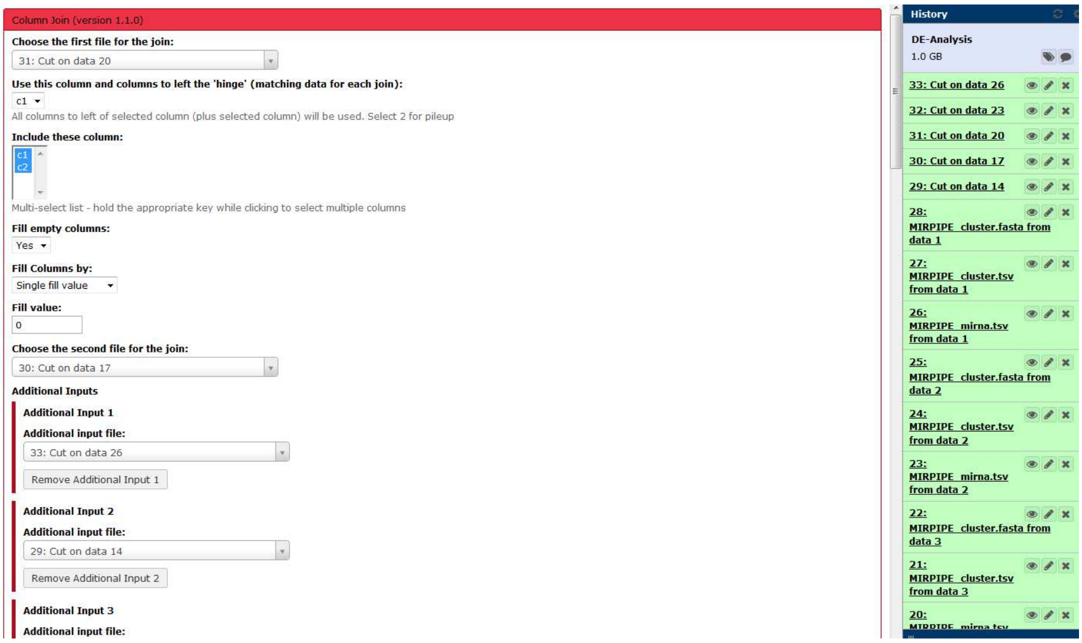

Figure 1: Joining MIRPIPE results into a matrix file.

Hint: You can order your inputs to the Column Join tool, since ordering is preserved. This means, that if you put replicates 1 and 2 from sample 1 as first inputs, they will occur as the first two columns in the count table. Subsequently, if you put replicates 1, 2 and 3 from sample 2 as another input, they will occur in this order in the count table.

Use the matrix file resulting from the Column Join as a count table for differential expression.

let-7	10434	42093	7274	4868	4906
let-7a	11674	46307	8390	5446	5548
let-7b	1145	6890	1210	598	849
let-7c	1189	5006	1075	578	643
let-7d	609	2685	712	399	447
let-7e	1473	4183	1130	686	571
let-7f	4396	16473	2781	2004	1811
let-7g	3744	13586	2779	2010	1777
let-7i	274	0	451	228	255
let-7k	1286	3567	923	488	470
lin-4	18	53	0	15	0

Figure 2: Count matrix from the Column Join tool (only first rows shown). Columns correspond to different samples/replicates.

3. Use DESeq2 for each matrix file separately. By subsequent addition of replicates to one of the two samples (named sample1 and sample2 in Figure 3) you have to follow the ordering of the samples/replicates in the count matrix.

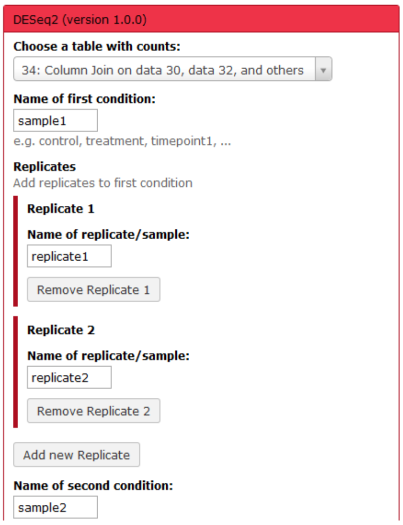

Figure 3: Input to the DESeq2 tool reflects the ordering of the count matrix.

4. The output will show a table with statistics (like mean, fold change and p-values) as well as an MA plot to depict the data. For detailed interpretation please consult the DESeq2 manual: http://www.bioconductor.org/packages/release/bioc/html/DESeq2.html.

6. CONTACT

Feel free to contact us with questions, bugs, suggestions, or anything else related to MIRPIPE.

Carsten Kuenne carsten.kuenne@mpi-bn.mpg.de
Jens Preussner jens.preussner@mpi-bn.mpg.de
Mario Looso mario.looso@mpi-bn.mpg.de

Max Planck Institute for Heat and Lung Research, Bad Nauheim, Germany
