# Supplementary material for: MIRPIPE: quantification of microRNAs in niche model organisms
Source: Bioinformatics. 2014 Aug 26;30(23):3412–3. doi: 10.1093/bioinformatics/btu573 (PMC4816158; doi:10.1093/bioinformatics/btu573)
Supplement: Supplementary Data [file supp_btu573_mirpipe_supplementary_2.pdf]

CONTENTS

1. Benchmark: MIRPIPE vs. published algorithms 1

2. Benchmark: MIRPIPE sensitivity with increasing reference distance 1

References 4

1. BENCHMARK: MIRPIPE VS. PUBLISHED ALGORITHMS

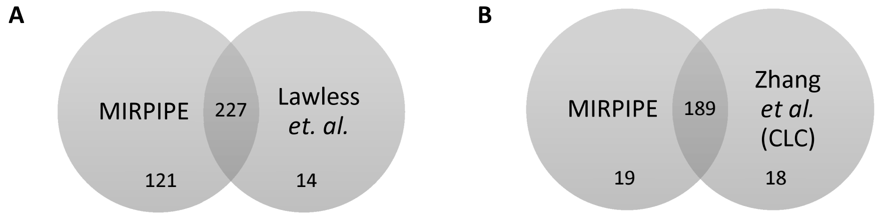

Figure 1. Comparison of detected miRNAs between MIRPIPE and other algorithms based on published data. A) depicts the overlap of identified miRNA families vs. a genome-based algorithm (Lawless et al., 2013). B) shows the overlap vs. CLC, a software which works similar to MIRPIPE by directly aligning against reference miRNAs (Zhang et al., 2013).

Two publications describing miRNA identification based on Illumina HiSeq 2000 RNASeq technology were processed and compared to MIRPIPE (Lawless, et al., 2013; Zhang, et al., 2013). Only miRNAs reaching a minimum read count of 5 were included to ward off spurious matches.

The publication by Lawless et al. used 450 million reads from bovine BME cells aligned versus the genome and then assigned to Ensemble v66 gene and miRNA annotations (Fig. 1A) (Lawless, et al., 2013). MIRPIPE was able to identify 96% out of 362 different miRNA families detected. One third of the miRNAs were identified by MIRPIPE but not by the algorithm used by Lawless et al. indicating that direct mapping to reference miRNAs results in increased sensitivity compared to an alignment of putative miRNA sequences to genome data of unproven quality.

Zhang et al. obtained 6 million sequence reads from mouse hearts (Fig. 1B) (Zhang, et al., 2013). We used these reads for identification of miRNAs by direct mapping to miRBase using CLC Genomics Workbench. Most of the miRNAs families detected by CLC (84%) were also identified by MIRPIPE indicating the competitiveness of the free MIRPIPE software compared to the commercial CLC package.

2. BENCHMARK: MIRPIPE SENSITIVITY WITH INCREASING REFERENCE DISTANCE

In order to prove the utility of our algorithm for niche model organisms lacking genome and reference miRNA sequences, we selected a human gold standard sample and treated it like a niche model. The dataset of human lymphoblastoid cells was sequenced using an Illumina HiSeq 2000 instrument (Lappalainen, et al., 2013). The

quantification values given by Lappalainen et al. are based on an algorithm including a genomic mapping step to weigh multi-mapped reads. We selected a random subset of 7 samples out of a pool of 462 individuals sequenced and chose two representatives for the manuscript. All 7 samples demonstrate similar patterns of expression/distribution and are included here for comparison. We run the MIRPIPE algorithm using the complete miRBase reference containing all human miRNAs for direct comparison. Furthermore we reduced the miRBase reference by removing human miRNA sequences and in a second step all primate miRNA sequences. This reduction served to simulate the effect of working in a niche model organism, lacking miRNA sequences from close relatives. The Venn diagrams in A) illustrate the effect of a reduced reference set to be negligible. Notably, miRNA predictions by Lappalainen et al. (Geuvadis) contain a large amount of low expression miRNAs that were filtered out by MIRPIPE default parameters (see column C).

<table>
<tr><td>A) Venn diagram of miRNA family distribution between Lappalainen et al. and MIRPIPE using various reference databases (complete, w/o human, w/o all primate)</td><td>B) Spearman correlation of miRNA family counts given by Lappalainen et al. (x-axis) versus a MIRPIPE run using the complete miRBase (y-axis)</td><td>C) Histogram of miRNA families expression values identified by Lappalainen et al. and not found by MIRPIPE</td></tr>
<tr><td>ERR187525
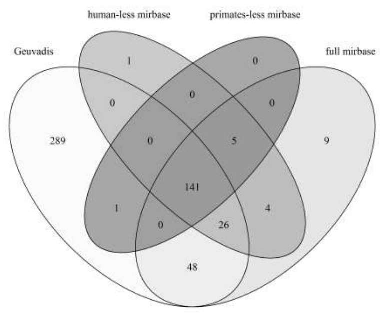
</td><td>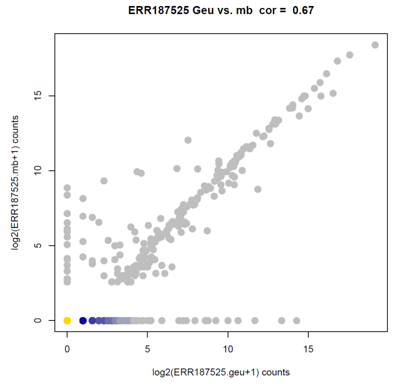
</td><td>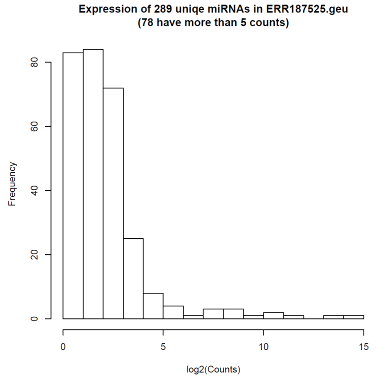
</td></tr>
<tr><td>ERR187597
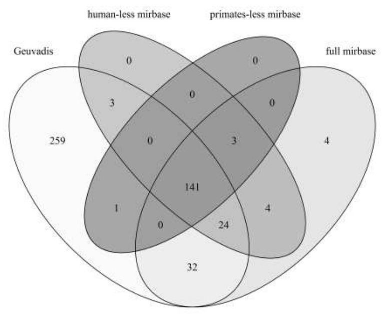
</td><td>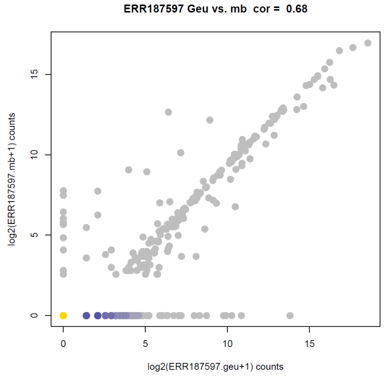
</td><td>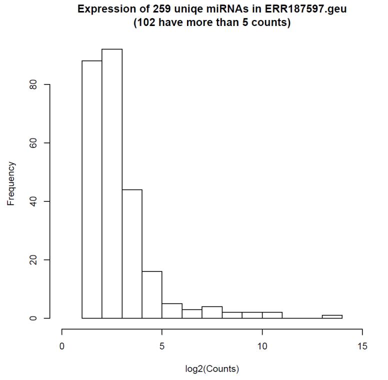
</td></tr>
</table>

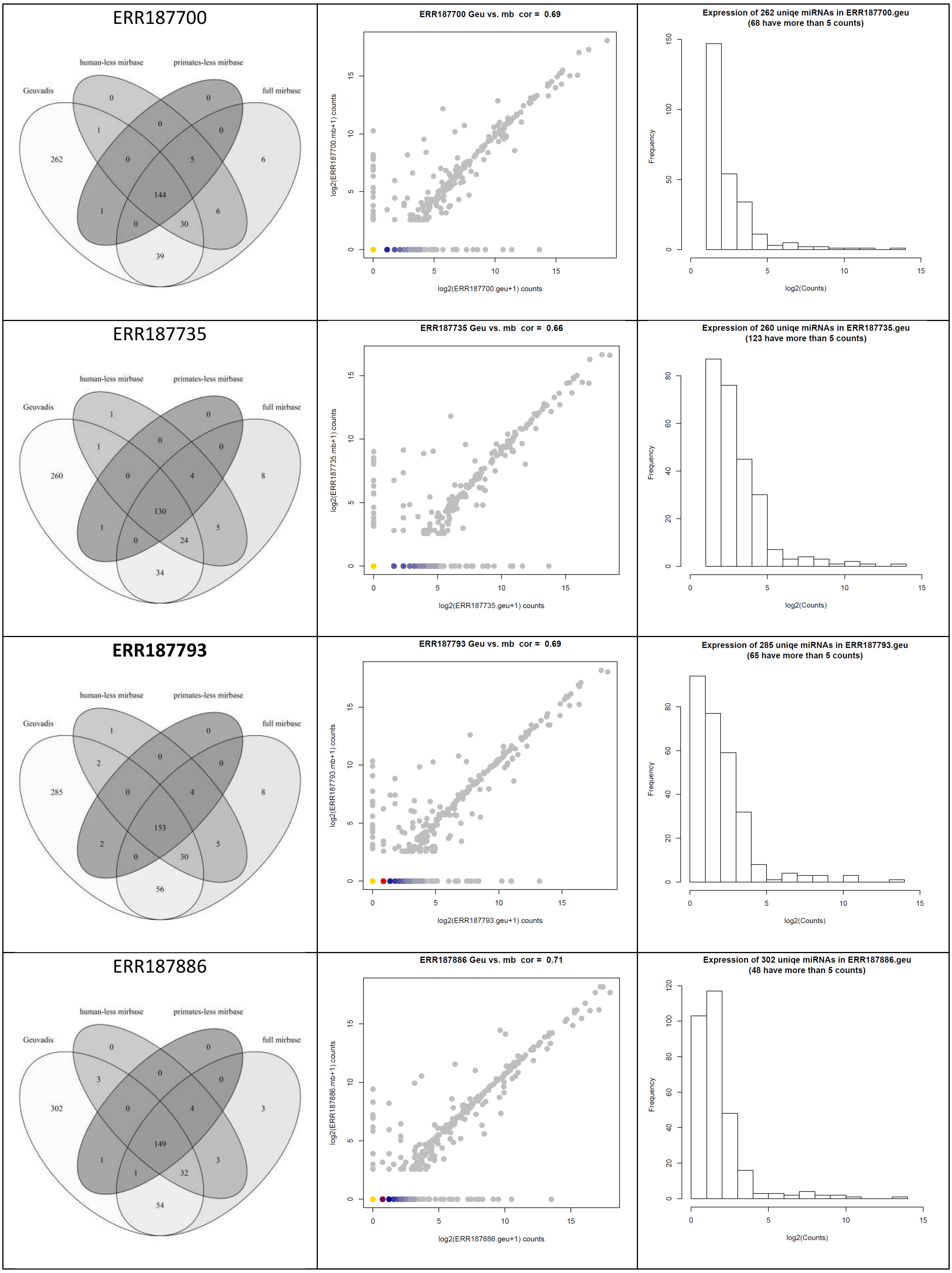
ERR187700
Geuvadis
human-less mirbase
primates-less mirbase
full mirbase
262
0
1
0
0
5
144
1
0
30
6
6
0
0
39
ERR187700 Geu vs. mb cor = 0.69
log2(ERR187700.mb+1) counts
log2(ERR187700.geu+1) counts
Expression of 262 uniqe miRNAs in ERR187700.geu
(68 have more than 5 counts)
Frequency
log2(Counts)
ERR187735
Geuvadis
human-less mirbase
primates-less mirbase
full mirbase
260
1
1
0
0
4
130
1
0
24
5
8
0
0
34
ERR187735 Geu vs. mb cor = 0.66
log2(ERR187735.mb+1) counts
log2(ERR187735.geu+1) counts
Expression of 260 uniqe miRNAs in ERR187735.geu
(123 have more than 5 counts)
Frequency
log2(Counts)
ERR187793
Geuvadis
human-less mirbase
primates-less mirbase
full mirbase
285
1
2
0
0
4
153
2
0
30
5
8
0
0
56
ERR187793 Geu vs. mb cor = 0.69
log2(ERR187793.mb+1) counts
log2(ERR187793.geu+1) counts
Expression of 285 uniqe miRNAs in ERR187793.geu
(65 have more than 5 counts)
Frequency
log2(Counts)
ERR187886
Geuvadis
human-less mirbase
primates-less mirbase
full mirbase
302
0
3
0
0
4
149
1
1
32
3
3
0
0
54
ERR187886 Geu vs. mb cor = 0.71
log2(ERR187886.mb+1) counts
log2(ERR187886.geu+1) counts
Expression of 302 uniqe miRNAs in ERR187886.geu
(48 have more than 5 counts)
Frequency
log2(Counts)

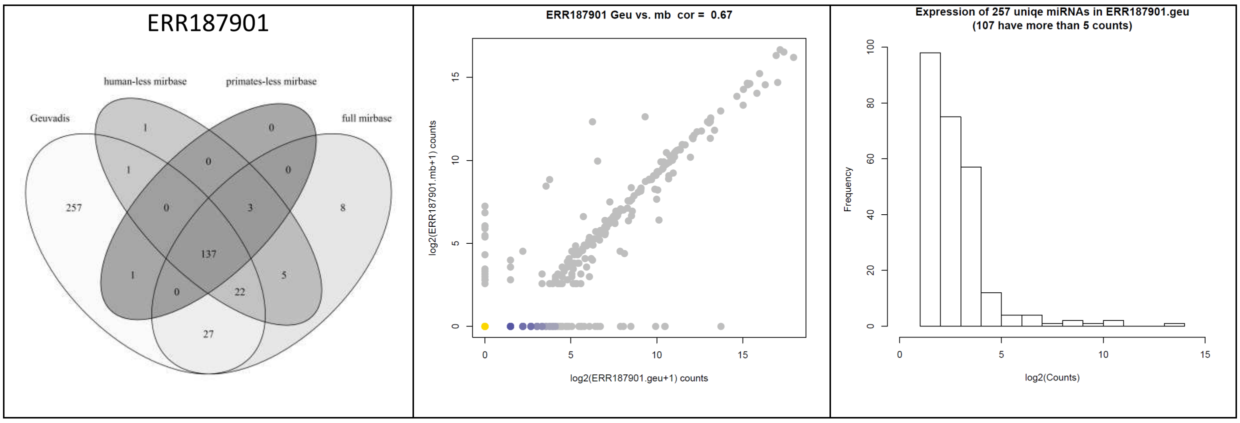

REFERENCES

Lappalainen, T., *et al.* Transcriptome and genome sequencing uncovers functional variation in humans. *Nature* 2013;501(7468):506-511.

Lawless, N., *et al.* Next generation sequencing reveals the expression of a unique miRNA profile in response to a gram-positive bacterial infection. *PloS one* 2013;8(3):e57543.

Zhang, Z., *et al.* High-efficiency RNA cloning enables accurate quantification of miRNA expression by deep sequencing. *Genome biology* 2013;14(10):R109.
